# The level of free circulating mitochondrial DNA in blood as predictor of death in case of acute coronary syndrome

**DOI:** 10.1186/s40001-016-0241-x

**Published:** 2017-01-03

**Authors:** Nikolay P. Sudakov, Konstantin A. Apartsin, Svetlana A. Lepekhova, Sergey B. Nikiforov, Alexander I. Katyshev, Galina I. Lifshits, Anna V. Vybivantseva, Yuri M. Konstantinov

**Affiliations:** 1 Irkutsk Scientific Center, Siberian Branch of the Russian Academy of Sciences, Lermontov St. 134, Irkutsk, 664033 Russia; 2 Irkutsk Scientific Center of Surgery and Traumatology, Bortsov Revolyutsii St. 1, Irkutsk, 664003 Russia; 3 Irkutsk State University, Karl Marx St. 1, Irkutsk, 664003 Russia; 4 Siberian Institute of Plant Physiology and Biochemistry, Siberian Branch of the Russian Academy of Sciences, Lermontov St. 132, Irkutsk, 664033 Russia; 5 Institute of Chemical Biology and Fundamental Medicine, Siberian Branch of the Russian Academy of Sciences, Lavrentiev Avenue 8, Novosibirsk, 630090 Russia

**Keywords:** Acute coronary syndrome, Myocardial ischemia, Mitochondrial DNA, Danger-associated molecular patterns

## Abstract

**Background:**

The efficacy of treating acute myocardial ischemic damages depends, to a large extent, on the development of technologies for predicting their course and outcome. The aim of this paper was to explore whether it would be possible to consider the content of free circulating mitochondrial DNA as a danger-associated molecular pattern for assessing the probability of death from myocardial infarction.

**Methods:**

We have analyzed the clinical outcomes based on discharge summaries and autopsy reports obtained in the course of the PROTOCOL observational trial. This study was approved by the Irkutsk Scientific Center of Surgery and Traumatology ethics committee (protocol No. 3, 10.08.2015). To examine whether the assessment of the level of free circulating mtDNA in acute coronary syndrome can help predicting clinical outcomes, all patients were divided into two groups: group 1, involving those who survived during 30 days after hospitalization, and group 2, involving those who died during this time. A quantitative analysis of the free circulating mtDNA was conducted using the PCR method in situ.

**Results:**

The analysis showed that in patients who survived the level of freely circulating mtDNA (36.0 copies/ml) was 164 times lower than in those who died (5900 copies/ml, *p* = 0.049). It should be mentioned that according to the logistic regression analysis, the probability of death of patients with the increased level of blood plasma mtDNA (more than 4000 copies/ml) is 50%.

**Conclusions:**

Thus, the PROTOCOL observational trial proved that the increase in the content of free circulating mtDNA in blood is a predictor of lethal outcome in patients with acute coronary syndrome.

*Trial registration* The observational studies (those in which the assignment of the medical intervention is not at the discretion of the investigator) do not require registration.

## Background

The development of technologies for predicting acute myocardial ischemic damages helps to increase the efficacy of treatment to a great extent. Currently, the so-called danger-associated molecular patterns (DAMPs) are considered to be predictors of adverse clinical outcomes and deaths from various diseases. DAMPs include nuclear and cytoplasmic macromolecules activating the components of the innate immune system after having been released from injured tissues [1]. One of the little-studied but promising DAMPs reflecting intense cytolytic processes is free circulating mitochondrial DNA (mtDNA). Today, the level of blood mtDNA is used to forecast complications and morbidity for malignant neoplasms and septic processes, and to assess the probability of death in emergency patients [2–4]. However, the dynamics of concentration of free circulating blood mtDNA in acute myocardial ischemic damages has not been explored yet. Therefore, it seems vital to examine the possibility of using this parameter as a biomarker of damaged cardiac muscle cells in comprehensive diagnosis of myocardial infarction. It was already explored the dynamics of the level of free circulating blood plasma mtDNA in acute myocardial ischemia and observed the increase in blood plasma mtDNA concentration [5–7]. Recently, we found the raise of concentration of free circulating mitochondrial DNA in blood after injection of adrenaline that caused the development of multiple small focal myocardial ischemia in Wistar rats [8, 9]. Recent observations [10] reveal the molecular mechanism behind the development of acute heart failure in animal models, where mitochondrial DNA is an important pathogenic factor. At high level of mtDNA in blood, it can cause inflammation and disorder of heart function [10]. The aim of this paper was to explore whether it would be possible to consider the content of free circulating mitochondrial DNA as a danger-associated molecular pattern for assessing the probability of death under acute coronary syndrome.

## Methods

### Design of the study

We included, in the study, the results of medical examination (non-interventional trial) of the patients who signed informed consents for processing their data in the course of the Personalized the Rapy with clOpidogrel during coronary sTent implantation for acute coronary syndrOme taking into aCcount genetic pOLymorphism (PROTOCOL) prospective multicenter observational study. (Organization conducting the study: Institute of Chemical Biology and Fundamental Medicine, SB RAS; Collaborators: Department of Medicobiological Studies and Technologies, Irkutsk Scientific Center, SB RAS, and Irkutsk Scientific Center of Surgery and Traumatology; Protocol Authors: Prof. K. A. Apartsin, Prof. G. I. Lifshitz, and N. Yu. Knauer; Study Director: Prof. G. I. Lifshitz, Doctor of Medicine, Head of the Laboratory of Personalized Medicine, Institute of Chemical Biology and Fundamental Medicine, SB RAS) [11]. This non-interventional trial was approved by the Irkutsk Scientific Center of Surgery and Traumatology ethics committee (protocol No. 3, 10.08.2015).

Emergency patients were admitted to the Irkutsk State Regional Hospital for percutaneous coronary intervention (PCI) by coronary stenting implantation (CSI) on account of in acute coronary syndrome (ACS). They were administered with load clopidogrel dose about 2 h before PCI, and afterwards, with daily maintenance dose of clopidogrel during 30 days after CSI. Three medical examinations (standard CSI trials) were conducted (48 ± 6 h after CSI; at the day of discharge from the hospital; and 30 ± 3 days after CSI) to assess the efficacy and safety endpoints [12]. According to the criteria of Academic Research Consortium, definite or probable stent thrombosis was registered as efficacy endpoint [13]. Heavy and medium clinically significant bleeding was regarded as safety endpoint. At stages 1‒3, the data were collected during medical examinations; at stage 4, as telephone survey.

#### Criteria for being included in the research

1. ACS diagnosis requiring urgent hospitalization and CSI;
2. Patient’s informed consent to participate in the observational study.

#### Criteria for non-inclusion

1. Recent administration of clopidogrel and/or anticoagulants (during the last 14 days);
2. Thrombolytic therapy at the pre-hospital stage;
3. Active (clinically significant) bleeding at the time of screening during the first examination (stage 1);
4. Clopidogrel intolerance in past medical history;
5. PCI without CSI (e.g., diagnostic procedure or balloon angioplasty);
6. Refusal of the patient (or of his legal representative in case if the patient cannot express his will) to participate in the study at any stage;
7. Incapability of the patient, according to the researcher, to interact (participate in the survey) during stage 4 held as telephone survey.

Clinical outcomes were analyzed based on discharge summaries and autopsy reports. In accordance with clinical judgment, two outcomes were distinguished: favorable progression after CSI (alleviation of pain syndrome, general recovery, and absence of signs of stent thrombosis), and unfavorable progression (death after CSI from heart failure, clinical symptoms of stent thrombosis, and progressive functional disorders). Blinding assessment of the course after CSI was conducted by an experienced cardiologist based on the data provided. We analyzed 14 enumerated test tubes and selected blood plasma samples of patients with favorable and unfavorable progressions after CSI. Appraisal sheets with clinical laboratory data of the patients and integral assessment (favorable or unfavorable progression after CSI) marked in accordance with the numbers on test tubes were sent for unblinding procedure conducted by an analyst evaluating the level of mitochondrial DNA. The unblinding procedure was carried out after the analysis of the level of freely circulating mitochondrial DNA had been done.

### Analysis of the level of free circulating blood mtDNA

**Fig. 1:**
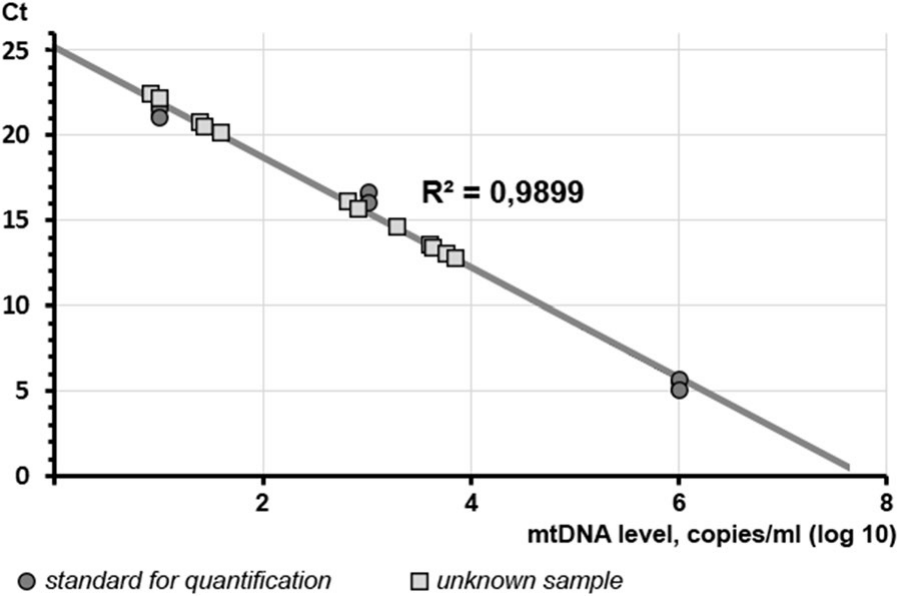
Calibration straight line. *Ct* threshold cycle

At stage 1, blood samples used for evaluating the concentration of mitochondrial DNA were collected not later than after 2 h upon the administration of the load clopidogrel dose; at stage 2, not later than after 48 ± 6 h after CSI. To analyze mitochondrial DNA, we collected platelet-poor plasma obtained by centrifugation of patient’s blood at 3000 rpm during 15 min. Isolation of total DNA from blood plasma was performed with PROBA-NK reagent kit (DNA-Technology, Russia) according to the manufacturer’s instruction. The quantitative analysis of mtDNA was conducted using PCR method in situ with the reaction mixture containing SYBR Green (Maxima^TM^ SYBR Green/ROX qPCR Master Mix–Thermo Fisher Scientific Inc., USA). The amplification of 16S rRNA gene fragment (length 230 base pairs) was performed (forward primer: 5′-CAGCCGCTATTAAAGGTTCG-3′; reverse primer: 5′-GGGCTCTGCCATCTTAACAA-3′) [2]. To draw a calibration straight line (Fig. 1), a set of samples with the precisely known concentration (10, 1000, and 1,000,000 copies/ml) of the above stated gene (length 490 base pairs) containing the analyzed sequence with the length of 230 base pairs was used (forward primer: 5′-GGGATAACAGCGCAATCCTA-3′; reverse primer: 5′-ACGTTGGGGCCTTTGCGTAG-3). Oligonucleotide synthesis was carried out by Syntol OOO (LLC) (Moscow, Russia). The DT lite amplifier (DNA-Technology, Russia) was used to perform PCR in situ.

### Experimental statistics

The results of the quantitative assessment were presented as a median (25–75 percentiles). We applied non-parametric statistical methods and conducted exploratory data analysis using Spearman’s rank correlation coefficient. Based on this analysis, we built a logistic regression model of death probability depending on mtDNA level in blood plasma. To calculate the statistical significance of differences in mtDNA level in survivors and deceased patients, the Mann–Whitney test criterion was used.

## Results and discussion

**Fig. 2:**
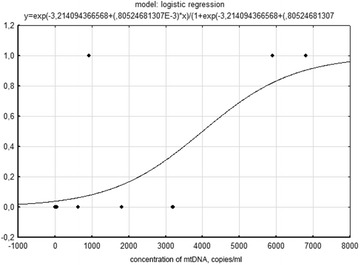
Logistic regression of death probability in patients with acute coronary syndrome depending on concentration of mitochondrial DNA upon arrival at the hospital

To examine whether the assessment of the level of free circulating mtDNA in acute coronary syndrome can help in predicting clinical outcomes, all unblinded patients were divided into two groups: group 1, involving those who survived during 30 days after hospitalization, and group 2, involving those who died due to ACS during this time. Therefore, equivalence in the ratio of deceased and survived patients is defined by the number of retrospectively included examination results and patients’ CSI, who signed informed consent for data handling within the Prospective Multicenter Observational Investigation PROTOCOL. Enlarging the group of deceased at the expense of patients not included into the PROTOCOL study would violate the standards of the present investigation. On the basis of the presented data, the blinding expert evaluation of the clinical course after CSI was conducted. Characteristics of the patients (sex, age, intercurrent diseases, and the level of blood plasma mtDNA) are presented in Table 1. In general, the ratio of survived and deceased patients roughly corresponds to the data of previously performed CSI trials [12]. Diabetes mellitus was observed in 60 and 67% of patients from groups 1 and 2, respectively. Essential hypertension was registered in group 1 (54.5% of patients) and group 2 (100%). An unfavorable course of acute coronary syndrome was observed in the patients of group 1 (30%) and group 2 (100%). The analysis showed the level of freely circulating blood mtDNA in those who survived was 164 times lower than in those who died (*p* = 0.049). The connection between the level of mtDNA and lethal outcome evaluated using the Spearman’s rank correlation coefficient (R2) is 0.4 (*p* = 0.01). At the same time, the logistic regression analysis of death probability in patients with acute coronary syndrome depending on concentration of mitochondrial blood plasma DNA upon arrival at the hospital indicates that the death probability of patients with increased mtDNA level (more than 4000 copies/ml) is 50% (Fig. 2). This conclusion is in agreement with the work by Nakahira [3] where the authors prove the possibility of regarding the level of free circulating mtDNA as predictor of death in emergency patients.

**Table 1:** General characteristics of patients included in the clinical study

	Survivors	Deceased	p
Number of patients	11	3	–
Age (years)	53.0 (52.0; 74)	87 (65.0; 87)	0.039
Sex (f)	2 (20%)	3 (100%)	0.035
Diabetes mellitus	6 (60%)	2 (67%)	1.000
Essential hypertension	6 (54.5%)	3 (100%)	0.496
Unfavorable course of ACS according to experts	3 (30%)	3 (100%)	0.070
Content of mtDNA (copies/ml)	36.0 (30.0; 1800)	5900 (920.0; 6800)	0.049

Recently [14], it was shown that plasma levels of mtDNA in elderly healthy are much higher than in young people. The authors measured mtDNA content in plasma from young (average age 36.8) and old (average age 82.7) trauma patients and corresponding young (average age 25) and old (average age 72) volunteers. Using young volunteers’ mtDNA levels as a control value of 100%, elderly volunteers, young trauma patients, and elderly trauma patients had values of 385, 364, and 2156, respectively [14].

The following observation made by us in the course of this study is worth mentioning and requires future investigation: only women turned out to be in the group of deceased patients after unblinding of mtDNA analysis results. At this stage of investigations, such a result could be possibly explained by the number of patients included into the PROTOCOL investigation. However, at present, we cannot fully exclude the existence of genetically determined association of patients’ gender with the blood plasma mtDNA content. Unfortunately, by now, we could not find any available published data on gender differences in the content of freely circulating blood mtDNA, though there exist data concerning the higher level of whole blood mtDNA of elderly women in comparison with men [15]. The discovered mtDNA level was determined by the number of platelets and white blood cells [16]. Taking this into consideration, we cannot use these data for discussion of the results in this study.

It is evident that to gain a deeper understanding of the reasons for such a drastic increase in the mtDNA content in patients with ACS, we need the data that are currently lacking as to the state and dynamics of changes in the structural and functional organization of mitochondrial membrane and cardiomyocyte cell membranes. Considering current knowledge of the biological nature of circulating nucleic acids [17], one can assume that the increase in mtDNA concentration above normal is caused by two consequent events: (i) release of mtDNA from mitochondria into cytoplasm, and (ii) release of mtDNA from cytoplasm of cells tissues and organs subjected to abnormally high stresses. The release of mtDNA from mitochondria into cytoplasmic space may occur both as a result of disruption of the continuity of mitochondrial membrane and of induction of mitochondrial permeability transition pore (MPTP) opening and/or potential reversibility of the earlier discovered mechanism of active DNA uptake by mitochondria [18, 19]. As for appearance of mtDNA in extracellular space, the possible mechanism here could be the release of mtDNA from the myocardial infiltrating cardiomyocytes and phagocytes dying by necrosis-like programmed cell death. Another possible way could be the release of mtDNA from cells with small membrane vesicles budding off from their surface [20]. Another mechanism of increasing the level of free blood mtDNA is their active release from cells caused by stress stimulus [21]. This process is mostly explored with regard to leukocytes releasing mtDNA included into the so-called extracellular traps [22]. Considering rapid changes of the mtDNA level, one cannot exclude the possibility that the drastic increase in the content of blood mtDNA in patients with ACS may be caused by the programmed mtDNA release from mitochondria and cells and, in turn, may serve as an additional regulatory factor for inducing massive apoptosis leading to death.

It is certain that comprehensive understanding of the processes at the molecular, cellular, and organ levels increasing the level of blood mtDNA will allow to reveal new cell targets for personalized therapy of patients with acute coronary syndrome. These data are important for purposes of translational medicine aimed at rapid introduction of the findings of fundamental studies to hospital practice [23].

## Conclusions

As a whole, the data obtained in the course of the PROTOCOL clinical study prove that increase in the content of freely circulating blood mtDNA up to a certain level is a clear predictor of lethal outcome in patients with ACS. This will help to forecast and influence the clinical course of myocardial infarction.

## Abbreviations

ACS: acute coronary syndrome
CSI: coronary stent implantation
DAMPs: danger-associated molecular patterns
mtDNA: mitochondrial DNA
PCI: percutaneous coronary intervention
